# Supplementary material for: Chromothripsis during telomere crisis is independent of NHEJ, and consistent with a replicative origin
Source: Genome Res. 2019 May;29(5):737–49. doi: 10.1101/gr.240705.118 (PMC6499312; doi:10.1101/gr.240705.118)
Supplement: Supplemental Material [file supp_gr.240705.118_Supplemental_file_1.zip › contigs/annotated_contigs/DB80/contig.2.DB80_length_614_mean_cov_10.5016286645.docx]

**DB80_length_614_mean_cov_10.5016286645**

```
TAGATTCTCACACTCAATTAAAAAAAGAAAGCCTGAGGAAATTAAATGATTTCTAAAATCACCCAGCTAATTAGTGGCAGAAATGAGAC
 >chr14:80201031-80201333 + E=5e-170
CAGATTCCAGTCTCTTGCTTTCCCGAATACCTTATTCTGGAATTCAATTATAAGTACAAGGCATCCCACTCTTGGCTTCCTTTCTGAAT

AAATGCTTGCACCAAATCCCCAGTGGCTTCTTGAGAAAGCTGGCTACAAATCTTCAAAGCCTCAGTTGAAGAATTCTTATTAAGCCTAC

TAGGTACCAAAGCAATAGCCTGAAGTTTGGAAAAA|AATTTGGT|TATATATATATATATATATATAACCTTACTTCTGCTTTTAAACA
                                            >chr14:80207089-80207393 + E=3e-168
TAGATCATTATTTCCATGTCTACCTTCCACTTTCTCCAACACTAGATTGCAAATTTCTACAGGGTGAAGCTTATGTTTTCATCCCTTAA

CACCCAGCTTAAATGTCACCTCACATACTGACTGATGCTTTTTATCTCAGTTCTAAAACATTATTAATGTACATCACATAATTTAACTC

CATTTTGAAAATTAGTTCTTGTTCCAGTATCTTATCTCTTAAAATAGACTGGAACCATCTCAAGGTTAGAGAGGATCCACTA
```
